# A multi-year (2007-2025) moth community dataset across the islands of southern South Korea

**DOI:** 10.3897/BDJ.14.e211519

**Published:** 2026-10-23

**Authors:** Sei-Woong Choi, Taeman Han, Chang-Gyu Park, So-Yun Jo, Jae-Young Lee

**Affiliations:** 1 Mokpo National University, Muan, Republic of Korea Mokpo National University Muan Republic of Korea; 2 Korea National Park Research Institute, Wonju, Republic of Korea Korea National Park Research Institute Wonju Republic of Korea

**Keywords:** Abundance, islands, Korea, moths, National Parks, species richness

## Abstract

**Background:**

Island ecosystems provide natural laboratories for understanding patterns of biodiversity, species turnover and the ecological processes shaping community assembly. Moths (Lepidoptera) are amongst the most diverse insect groups and play important ecological roles as herbivores, pollinators and prey for higher trophic levels, making them valuable indicators of environmental change. Although numerous ecological studies have examined moth diversity in continental forests, standardised long-term datasets spanning multiple islands remain scarce, particularly in East Asia. To facilitate studies of island biogeography, temporal community dynamics and biodiversity conservation, we compiled a standardised dataset of moth community composition from islands along the southern coast of the Korean Peninsula.

**New information:**

This dataset contains standardised light-trap survey records collected from 332 sites on 30 islands between 2007 and 2025, encompassing 1,179 moth species from 19 families and 30,442 individuals. Most islands are located within two marine national parks, providing broad coverage across gradients of island area, isolation and elevation. The dataset includes site-level species abundance matrices, island-level environmental variables (area, distance from the mainland and maximum elevation), survey metadata and taxonomic information for all recorded species. Each island was surveyed in one to four discrete years, enabling analyses of both spatial variation amongst islands and temporal variation within islands. This dataset provides a valuable resource for studies of island biogeography, community assembly, biodiversity monitoring, environmental change and conservation prioritisation of insular insect communities.

## Introduction

Islands can be broadly defined as spatial units that are physically or ecologically isolated from their surroundings by water barriers, topographic features or habitat fragmentation. The concept, therefore, extends beyond marine environments to include mountaintops, lakes and terrestrial habitat fragments. Marine islands, defined here as landmasses surrounded by seawater, constitute a particularly important group in ecological and biogeographic research. They include volcanic oceanic islands, coral atolls, and islands on continental shelves, including those formerly connected to the mainland (MacArthur and Wilson 1967, Gillespie and Roderick 2002). These island types differ substantially in geological origin, age and degree of isolation. Such differences shape ecological processes, species dispersal and biogeographic patterns, contributing to the diversity of island habitats and their associated flora and fauna (Kotze et al. 2000,Gillespie and Roderick 2002, Weigelt et al. 2016, Whittaker et al. 2017, Fernández-Palacios et al. 2021). In particular, continental islands on continental shelves are often less isolated than remote oceanic islands and provide valuable opportunities to examine how connectivity and proximity to mainland species pools influence ecological dynamics (Kotze et al. 2000, Weigelt et al. 2016).

The ecological significance of islands is reflected in their disproportionate contribution to global biodiversity: they support an estimated 15–20% of terrestrial biodiversity, while occupying only 6.67% of Earth’s land area (Kier et al. 2009; Sayre et al. 2018; Fernández-Palacios et al. 2021). Distributed across all ocean basins, marine islands vary widely in size and distance from the mainland, although small islands predominate numerically. The Global Island Database lists approximately 175,000 islands with areas of 0.1–1 km² and 17,883 islands larger than 1 km². In Korea, there are 3,390 marine islands, of which 480 (14.2%) are inhabited and the remainder are uninhabited (Anonymous 2021, Korea Island Development Institute 2026). Most uninhabited islands are small and situated near the mainland: approximately 93.8% of them are smaller than 0.1 km² and 84.8% (2,474 islands) lie within 20 km of the mainland. These predominantly small, nearshore islands provide a geographical context for investigating island biodiversity in relation to mainland proximity.

Island biotas have been shaped by long-term evolutionary and ecological processes following their formation or isolation from the mainland. Interactions amongst geographic, ecological, biological and temporal factors have generated unique communities with high levels of endemism (Vitousek et al. 1995, Whittaker and Fernández-Palacios 2007, Lomolino et al. 2010), yet this exceptional biodiversity remains highly vulnerable to both natural and human-induced disturbances (Russell and Kueffer 2019, Fernández-Palacios et al. 2021).

Moths are one of the most taxonomically diverse insect groups, comprising more than 160,000 described species worldwide. They play key ecological roles as herbivores, pollinators and prey for a wide range of vertebrates, thereby contributing to ecosystem functioning and trophic interactions. Their high species diversity, efficient sampling using light traps and close associations with environmental conditions also make moths valuable indicators for biodiversity monitoring and environmental change (Wagner et al. 2021).

Here, we present a dataset on the richness and distribution of 1,179 moth species, representing 41.4% of all species known from Korea within the 19 moth families investigated. The dataset was compiled from surveys conducted between 2007 and 2025 on 30 islands in southern South Korea, comprising 28 islands within Dadohaehaesang and Hallyeohaesang National Parks and two islands outside these parks, including Gageodo, the southwesternmost island of South Korea. Dadohaehaesang National Park covers 2,266.221 km² and encompasses 400 islands, whereas Hallyeohaesang National Park covers 537.479 km² and encompasses 108 islands.

**Figure 1.**
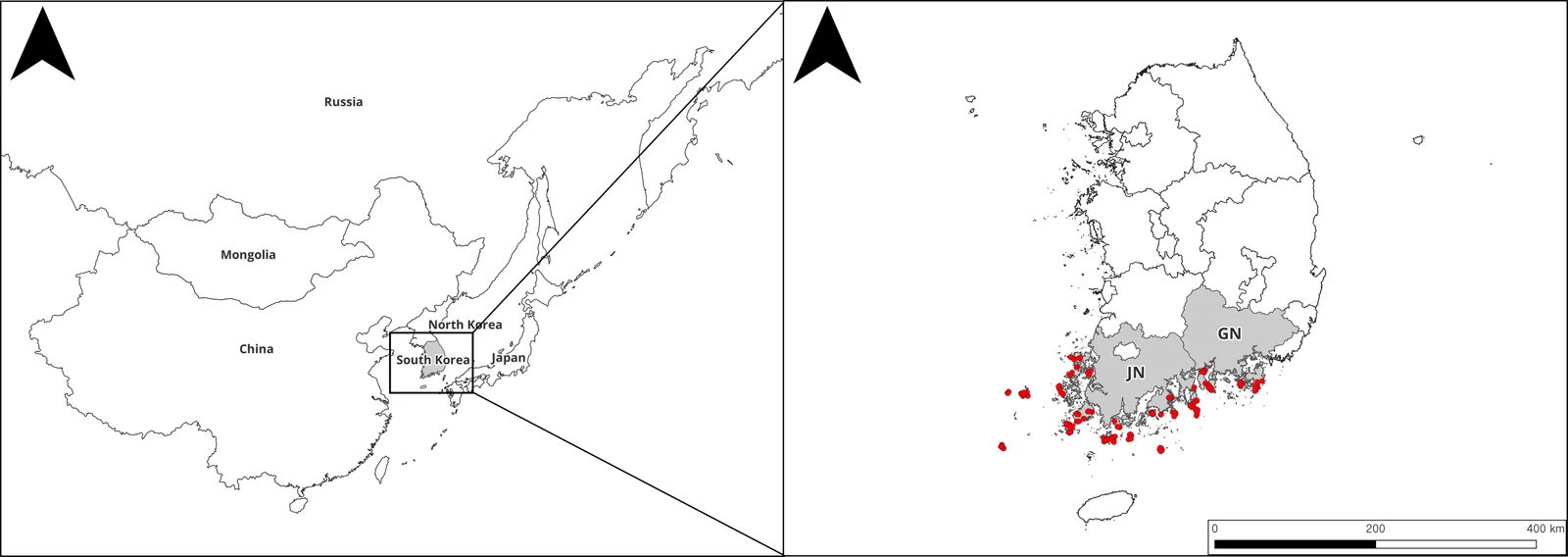
Study sites for moth sampling in southern South Korea. Red dots indicate the surveyed islands.

Sampling was conducted using ultraviolet (UV) light traps at 332 sites across coniferous, evergreen and mixed coniferous–deciduous forests (Fig. 1). Sampling coverage varied amongst islands and years (Tables 1, 2).

## General description

### Additional information

Between 2007 and 2025, standardised moth surveys were conducted across 30 islands, mostly within the Dadohaehaesang and Hallyeohaesang National Parks of South Korea. The dataset comprises 332 survey sites and documents 1,179 moth species, representing one of the most comprehensive long-term island moth datasets available for Korea. Survey intensity varied amongst years because field investigations followed the national biodiversity monitoring programme, under which many protected islands are surveyed at approximately five-year intervals. Consequently, the number of islands surveyed each year ranged from 1 to 10 and the annual sampling effort ranged from 1 to 65 survey sites (Table 1). Nineteen islands were surveyed in two or more years, contributing species occurrence records from multiple years, whereas eleven islands were surveyed in a single year, extending the spatial coverage of the dataset. These repeated surveys do not necessarily constitute standardised temporal replicates and differences in sampling coverage limit direct comparisons of community composition amongst years (Table 2).

Species richness differed substantially amongst islands, reflecting differences in island size, habitat diversity and survey history. Namhae Island yielded the highest cumulative richness (670 species), followed by Bogildo (345 species), Oenarodo (339 species), Geojaedo (337 species) and Geogumdo (312 species), whereas smaller or less intensively surveyed islands generally supported fewer recorded species (Table 2). Species richness generally increased on larger islands and on islands surveyed repeatedly across multiple years, although survey history also contributed to variation in the cumulative number of recorded species. Here, we present one of the most comprehensive long-term datasets on island moth assemblages available for South Korea, containing records of 1,179 species across 19 families. These species represent 41.4% of all species known from Korea within the families investigated.

This dataset provides a valuable resource for addressing a wide range of ecological and biogeographical questions in island ecosystems. Integrating species occurrence and abundance data across multiple islands enables rigorous evaluation of how island characteristics, such as area, isolation and elevational heterogeneity, influence biodiversity patterns and community structure. The dataset is, therefore, well suited for testing predictions of island biogeography theory and other macroecological frameworks.

The broad spatial (two marine national parks across southern South Korea) and temporal coverage (2007-2025) also allows investigation of species’ ecological niches, habitat associations and geographic distributions, providing opportunities to quantify niche breadth and assess species-specific responses to environmental variation and climate change. Time-resolved occurrence records further support phenological analyses by enabling assessments of seasonal activity patterns and long-term shifts in species occurrence.

At the community level, the dataset facilitates analyses of both spatial and temporal beta diversity, allowing researchers to quantify species turnover, nestedness, and community re-assembly across islands and years. These applications can improve our understanding of the relative roles of environmental filtering, dispersal limitation and stochastic processes in shaping island communities.

Beyond fundamental ecological research, the dataset provides an important baseline for long-term biodiversity monitoring and conservation planning. It can support regional and national species inventories, checklists and Red List assessments, while also helping identify vulnerable island ecosystems, evaluate community resilience and provide information for evidence-based conservation and management strategies under ongoing environmental change.

## Sampling methods

### Study extent

We focused on moths from the following 19 families: Cossidae, Zygaenidae, Limacodidae, Thyrididae, Crambidae, Pyralidae, Erebidae, Nolidae, Noctuidae, Eutelidae, Notodontidae, Geometridae, Drepanidae, Uraniidae, Saturniidae, Sphingidae, Lasiocampidae, Bombycidae, and Brahmaeidae. To assess the moth community, we placed one portable UV light trap at each survey point. Moths were attracted to the traps, equipped with a single 22-watt UV light (BioQuip Co.), powered by 7.2 Ah/12 V lead-acid batteries, and euthanised with dichlorvos. Traps were working from dusk for 5 hours under suitable weather conditions for moth trapping, avoiding heavy rain, strong winds and full moons. The samples were stored in paper bags and maintained at −22°C prior to species identification. The identification used in this study follows the literature (Kononenko et al. 1998, Kim et al. 2001, Shin 2001, Kishida 2011, Kim et al. 2016).

### Sampling description

Field sampling constraints and logistics resulted in uneven temporal coverage, which is explicitly documented and accounted for in the dataset. In South Korea, comprehensive biodiversity surveys in protected areas such as National Parks are legally conducted at five-year intervals, meaning that some islands were surveyed only during a single assessment period. Thus, sampling effort varied amongst islands and years, with some islands having data available for only a single year. All fieldwork was conducted in accordance with the applicable legislation of Korea. We obtained the collection and entry permits required for research within the Maritime National Parks.

### Quality control

Data processing was conducted to ensure taxonomic consistency, data quality and analytical reproducibility. Species names were standardised following the Korea Taxonomic Serial Number (National Institute of Biological Resources, https://species.nibr.go.kr), and synonymous or outdated names (e.g., Korean names) were revised. Records with ambiguous identifications or unresolved taxonomy were excluded. Occurrence records were then screened for data quality. Duplicate entries and records with missing or erroneous geographic information were removed. The final dataset consisted of 1179 species across 332 sampling sites, with associated information on taxonomy and geographic response categories.

### Step description

All sampling was conducted using standardised protocols across sites and years to ensure comparability. UV light trap deployment, sampling duration (5 hours after dusk), and seasonal timing (from spring to autumn) were kept as consistent as possible. Species identification was primarily conducted by the first author using established taxonomic keys and the literature. Where necessary, identifications were confirmed by the genitalia examination and DNA barcoding using mitochondrial Cytochrome oxidase subunit I (COI).

Raw data were screened for inconsistencies, including taxonomic changes, missing values and duplicate records. All species abundance data were converted to a numeric format and validated prior to analysis. Sites with no recorded individuals were checked to avoid bias in beta-diversity calculations. Rare species were also included and evaluated for their influence on dissimilarity metrics.

## Geographic coverage

### Description

Thirty marine islands in southern South Korea, including the south-westernmost island, Gageodo and two maritime national parks, Dadohaehaesang and Hallyeohaesang National Parks.

## Taxonomic coverage

### Description

Cossidae, Zygaenidae, Limacodidae, Thyrididae, Crambidae, Pyralidae, Erebidae, Nolidae, Noctuidae, Eutelidae, Notodontidae, Geometridae, Drepanidae, Uraniidae, Saturniidae, Sphingidae, Lasiocampidae, Bombycidae and Brahmaeidae

## Temporal coverage

### Notes

2007-2025

## Usage licence

### Usage licence

Creative Commons Public Domain Waiver (CC-Zero)

## Data resources

### Data package title

Island moth community dataset 

### Resource link

https://doi.org/10.5281/zenodo.21895989

### Number of data sets

4

### Data set 1.

#### Data set name

Site-by-species community matrix

#### Description

'island': island identifier (30 unique islands); 'year': sampling year; 'sites': site identifier (332 unique sites across all islands/years; 'site_id' in 'data3' corresponds to this column); 'sp1'–'sp1179': species abundance values (integer counts per sampling unit; observed range 0–186 in the current file; no missing cells). Each row represents one sampling site surveyed in a given year (island × year × site) combination.

### Data set 2.

#### Data set name

Taxonomic information for each species column

#### Description

'Species id': matches the 'sp1'–'sp1179' column names in 'data1_islands_species_occurrences' (1,179 rows, 1:1 correspondence); 'Scientific name' - 'Family' - 'Host plant' (many entries are "unknown").

### Data set 3.

#### Data set name

Geographic information for each sampling site

#### Description

'island': island identifier; 'site_id': corresponds directly to the 'sites' column in 'data1-islands_species_occurrences'; 'Latitude', 'Longitude': decimal degrees; 'Elevation': site-level elevation (m) - distinct from the island-level maximum elevation reported in 'data4-island_statistics'.

### Data set 4.

#### Data set name

Island-level environmental variables

#### Description

'island': island identifier; 'distance': distance from mainland (km); 'area': island area (km²); 'elevation': island maximum elevation (m).

## Figures and Tables

**Table 1.** Annual survey history of island moth monitoring in South Korea, including the number of surveyed islands and sampling sites in South Korea (2007-2025).

**Year**	**No. of islands**	**Islands**	**No. of sites**
**2007**	1	Oenarodo	1
**2008**	1	Bogildo	5
**2009**	1	Gageodo	16
**2010**	4	Cheongsando, Gumodo, Hajodo, Heuksando	16
**2011**	4	Cheongsando, Gumodo, Hajodo, Heuksando	16
**2012**	1	Gageodo	14
**2014**	3	Geojaedo, Namhae, Odongdo	19
**2015**	2	Geogumdo, Wando	10
**2016**	1	Jindo	2
**2017**	10	Ando, Bogildo, Cheongsando, Geomundo, Gumodo, Jijukdo, Oenarodo, Soando, Wando, Yeondo	65
**2018**	10	Bigeumdo, Dochodo, Gwanmaedo, Hajodo, Heuksando, Hongdo, Jangdo, Jindo, Sangjodo, Youngsando	53
**2020**	7	Aphaedo, Imjado, Jaeundo, Jeungdo, Jido, Namhae, Odongdo	34
**2021**	8	Ando, Bogildo, Cheongsando, Geogumdo, Gumodo, Oenarodo, Soando, Wando	32
**2022**	8	Bigeumdo, Dochodo, Gageodo, Gwanmaedo, Hajodo, Heuksando, Hongdo, Sangjodo	45
**2023**	1	Jindo	8
**2024**	4	Hajodo, Jindo, Sangjodo, Wando	18
**2025**	2	Namhae, Odongdo	23

**Table 2.** Survey history and recorded species richness of 30 islands in South Korea, comprising 28 islands within Dadohaehaesang and Hallyeohaesang National Parks and two islands outside these parks, during 2007–2025. Numbers in parentheses indicate the number of sites surveyed in each year. Annual species richness represents the number of distinct species recorded across all sites surveyed on each island in that year. For each island, the “Total” row reports the cumulative number of unique sampling sites (in parentheses) and distinct species across all survey years, with each site and species counted once. Sampling coverage varied amongst islands and years; therefore, recorded species richness should not be interpreted as a standardised measure for direct comparisons.

**National Park**	**County**	**Island**	**Survey year (No. of sites)**	**Species richness**
**Dadohaehaesang**	Shinan	Bigeumdo	2018 (7)	73
			2022 (4)	107
			Total (11)	156
	Shinan	Dochodo	2018 (6)	66
			2022 (4)	141
			Total (10)	181
	Shinan	Youngsando	2018 (4)	79
			Total (4)	79
	Shinan	Heuksando	2010 (4)	66
			2011 (4)	45
			2018 (5)	124
			2022 (9)	107
			Total (18)	255
	Shinan	Hongdo	2018 (4)	49
			2022 (7)	54
			Total (11)	89
	Shinan	Imjado	2020 (3)	87
			Total (3)	87
	Shinan	Jaeundo	2020 (4)	148
			Total (4)	148
	Shinan	Jangdo	2018 (4)	112
			Total (4)	112
	Shinan	Jeungdo	2020 (2)	65
			Total (2)	65
	Shinan	Jido	2020 (3)	67
			Total (3)	67
	Yeosu	Gumodo	2010 (4)	34
			2011 (4)	48
			2017 (11)	128
			2021 (3)	72
			Total (18)	211
	Yeosu	Geomundo	2017 (9)	81
			Total (9)	81
	Yeosu	Ando	2017 (3)	57
			2021 (1)	15
			Total (4)	67
	Yeosu	Yeondo	2017 (8)	83
			Total (8)	83
	Wando	Wando	2015 (2)	182
			2017 (5)	100
			2021 (3)	98
			2024 (1)	1
			Total (10)	285
	Wando	Bogildo	2008 (5)	255
			2017 (7)	134
			2021 (5)	75
			Total (15)	345
	Wando	Soando	2017 (6)	112
			2021 (3)	29
			Total (9)	132
	Wando	Cheongsando	2010 (4)	57
			2011 (4)	179
			2017 (5)	128
			2021 (3)	29
			Total (12)	281
	Goheung	Oenarodo	2007 (1)	230
			2017 (6)	131
			2021 (2)	107
			Total (9)	339
	Goheung	Jijukdo	2017 (5)	123
			Total (5)	123
	Goheung	Geogumdo	2015 (8)	156
			2021 (12)	228
			Total (19)	312
	Jindo	Jindo	2016 (2)	30
			2018 (3)	8
			2023 (8)	150
			2024 (9)	86
			Total (16)	229
	Jindo	Gwanmaedo	2018 (10)	121
			2022 (5)	47
			Total (14)	155
	Jindo	Hajodo	2010 (4)	37
			2011 (4)	37
			2018 (5)	84
			2022 (4)	44
			2024 (4)	43
			Total (15)	194
	Jindo	Sangjodo	2018 (5)	74
			2022 (4)	77
			2024 (4)	56
			Total (10)	175
**Hallyeohaesang**	Namhae	Namhae	2014 (6)	189
			2020 (17)	337
			2025 (20)	534
			Total (35)	670
	Geoje	Geojaedo	2014 (10)	337
			Total (10)	337
	Yeosu	Odongdo	2014 (3)	49
			2020 (3)	49
			2025 (3)	65
			Total (6)	138
**Outside National Parks**	Shinan	Gageodo	2009 (16)	93
			2012 (14)	125
			2022 (8)	101
			Total (36)	227
	Shinan	Aphaedo	2020 (2)	90
			Total (2)	90
**Total**		**30 islands**	**332 sites**	**1,179**
